# A German Trial against Dentists for Using the Title "Doctor of Dental Surgery,"

**Published:** 1885-09

**Authors:** George C. Gaston


ARTICLE III.

### A GERMAN TRIAL AGAINST DENTISTS FOR USING THE TITLE "DOCTOR OF DENTAL SURGERY."

[Translated for AMERICAN JOURNAL OF DENTAL SCIENCE.]

BY GEORGE C. GASTON.

IN THE NAME OF THE KING!

In the suit against the dentists, Dr. Schunk & Company, (Dr. Spahn), both at Frankfort-on-the-Main, for offense against the Industrial Ordinance.

The first instance of the Royal County Court, of

Frankfort-on-the-Main, has decided in a sitting of the court on June 15th, 1885, which was participated in by Dr. Koerner, President of the County Court; Dr. Kienitz, Fechner, Associate Judges of the County Court; Dove, County Judge; Brodman, Assistant Judge, as judges; Frehsee, Chief States Attorney, representing the State; Auerbach, as Court Stenographer.

On the appeal of the Royal States Attorney against the decision of the Royal City Court, of Frankfort-on-the-Main, dated April 16th and May 7th, 1885, that the accused: Dentist, Christian Carl Schunk, born on the 27th of September, 1847, at Buedingen, (Protestant); Dentist, George Stuckert, born May 4th, 1845, at Groskarben, (Protestant); Dentist, Valentine Schmidt, born December 7th, 1846, at Eppingen, in Baiden, (Catholic); Dentist, Oscar Wendler, born November 24th, 1857, at Berlin, (Protestant); Dentist, Michael Joseph Spahn, born January 2nd, 1848, at Carlstadt, (Catholic); all residing at Frankfort-on-the-Main, are guilty of a violation of §147—No. 3—of the Industrial Ordinance, and are sentenced to a fine of 15 marks and costs, for which, in case of inability to pay, one day's confinement to be substituted for each 5 marks.

By judgment of the Royal City Court, of the 16th of April, 1885, the defendants Schunk, Stuckert, Schmidt and Wendler, and by judgment of the same court, May 7th, 1885, the defendant Spahn, have been found not guilty and acquitted of a violation of §147—No. 3—of the Industrial Ordinance. The Royal States Attorney took appeal from both of these decisions by brief, within the time allowed by law, on the 16th and 17th of April, and 7th and 9th of May, basing his appeal on the State Process Ordidance §§154–355–358.

Both decisions, being nearly of the same tenor and both being defandant mainly on the same points of law, and the appeal in both cases being homogenous, the court has ordered, according to §236 of the State Process Ordi nance, that both cases be taken up jointly.

The defendants and Counseller-at-Law, Dr. Geiger, as legal representative of defendant Spahn, have admitted, in accordance with the facts stated by the judge of the first instance, that they have designated themselves in newspaper advertisements and on business signs, as "Doctors of Dental Surgery," but do not consider themselves to have violated the law, having appended an explanation to the above advertisements, removing any impression of having received their degree in this country, this explanation consists in the cases of Schunk, Stuckert, Schmidt and Wendler in these words: "Graduated in America," respectively "Graduated in New York," Schunk and Schmidt abbreviating these words in the following manner: "Gr'd't in Amer.," while Spahn's advertisement in the *General Anzeiger* reads "American Doctor of Dental Surgery."

§147—No. 3 of the revised Industrial Ordinance, which the State considers violated by the action of the defendant's, holds everybody liable who, not having passed the state examination, designate themselves as "Doctor," (Surgeon, Oculist, Accoucheur, Dentist, Veterinary Surgeon), or using similar titles, by which the impression would be conveyed, that the so designated person had passed the required medical examination. In order to understand this law, the word "Graduation" must be defined as laid down in §29 of the Industrial Ordinance. In the regulation of the medical profession, &c., the right of an unrestricted following of a trade or profession, has not been set aside, as the proof of competence and fitness (with the sole exception of the profession of druggist) is not a condition *sine qua non*. But the law has awarded to those who pass an examination as required by the Ordinances of the Federal Council, a double privilege, first, by designating to such graduates certain official functions, (Circuit Veterinary Surgeon, Circuit Surgeon, &c.), and secondly, in allowing such graduates the use of certain titles which are regarded by the people as proof of having passed the examinations required by law. The titles Doctor, Surgeon,

Oculist, Accoucheur, Veterinary Surgeon have been fixed as such designations. §29 of the R. I. O. The laws of the United States do not conform with ours, as the *so-called* diplomas are not issued by a State authority, but by the purely private dental colleges.

The defense contends that by use of the words used in addition to the word "Dentist," a perfect and full explanation was made to the public, that their degree had been received in a foreign country and not in this State. But this cannot stand before the law, the defendants refer to the high state of dental technique in America, and this is a well-known fact, but they make a boast of having received their *so-called* "American degrees," as though the acquirance of a degree in this State was of no value whatever. If, however, the state of American dental technique is really of such a high order, then, surely very often dentists who have received their degree in Germany, would take another course in American colleges, and therefore the designation "American Dentist" may be construed as having received a degree in both countries, but apart from this the designation "Dentist" with or without explanatory additions, is contrary to law, the title "Dentist" as above stated, being a prerogative of those who have received a degree in this State, if used by others, is a deception of the public. The dental technique of America may be developed to the highest degree, but the *so-called* "American diploma" gives no guarantee that the graduate has followed the study diligently, not to say, has reached the highest perfection in it, and the traffic in totally worthless academical diplomas and titles, has also reached a very high degree in the United States of America, and therefore the law of this State requires that the possessors of such titles give a guarantee of their competency by proving to the authorities of this State, that they are equal to the standard required in this country. The question of the protection of foreign degrees can not be raised here, the question here is the protection of *home* degrees against deception. The defendants may have come up to the standard of the *so-called* American degrees, but in using titles only intended for persons graduated in Germany, they intend to convey to the public mind the impression that they also belong to such a privileged class.

In regard to the facts, the court has come to the conclusion that the defendants were well aware of the fact that by using the title "Dentist" they committed a deception of the public, it must also be further taken into consideration that a controversy in professional circles in regard to the right of using the title "Dentist" has been going on for a long time, and that numerous convictions have taken place. Even if they believe that by the use of additional words, they could circumvent the law, and thereby, perhaps elude punishment; they were at least conscious of the fact that they were acting in fraudem legis, the knowledge of this fact cannot be denied by the defendant Spahn, who declares that he asked Police Justice Genolla, and was informed by him that the use of the title "American Dentist" was proper. But the fact of his making the inquiry was evidence in itself that he was in doubt of the legality of his making use of the same. The information received by him can only be regarded as the expression of the private opinion of the Official, and as such it carried in itself no protection for the defendant, the defendant's have *intentionally* committed an illegal act, and therefore they cannot be protected by the plea of a misunderstanding of the law.

The fact was proved that the defendants at Frankfort-on-the-Main have described themselves as dentists during the first 3 months of the year 1885, without having received the proper degrees. Judgment was given according to §147—No. 3 of the I. O. In pronouncing judgment it must be taken into consideration that these are the first cases of the kind before this court in this city and therefore they may be regarded as test cases, in consideration of the circumstances each defendant has to pay a fine of 15 marks,

(about 4 dollars), in case of inability to pay—one day's confinement for each 5 marks to be substituted.

*Signed:* DR. KOERNER,
" v KIENITZ,
" FECHNER,
" DOVE,
" BRODMAN.

*Witness:* PAEHLER,
Court Stenographer.

